# Special Issue “Materials for Photobiology”

**DOI:** 10.3390/ijms25063209

**Published:** 2024-03-12

**Authors:** Angela Scala, Enrico Caruso, Antonino Mazzaglia

**Affiliations:** 1 Department of Chemical, Biological, Pharmaceutical and Environmental Sciences, University of Messina, V.le, F. Stagno d’Alcontres, 31, 98166 Messina, Italy; 2 Department of Biotechnologies and Life Sciences, University of Insubria, Via JH Dunant 3, 21100 Varese, Italy; enrico.caruso@uninsubria.it; 3 National Council of Research, Institute for the Study of Nanostructured Materials (CNR-ISMN), URT of Messina c/o Department of Chemical, Biological, Pharmaceutical and Environmental Sciences, University of Messina, V.le, F. Stagno d’Alcontres, 31, 98166 Messina, Italy

Photobiology is a challenging research area that aims to explore the interactions between light and living organisms and their biological consequences, with applications in the fields of photomedicine, photo(nano)technology, photosynthesis, and photosensory biology [1]. Nowadays, there is great interest in designing advanced materials with peculiar physicochemical properties and photoresponsive features that, through interactions with light, produce a response which can be exploited for diagnosis and/or therapy [2]. Therefore, the interaction of light with molecules, nanomaterials, cells, and tissues and the subsequent responses elicited represents a multidisciplinary field of research bringing together chemists, physicists, biologists, biochemists, medical specialists, and many others.

In this Special Issue, articles and reviews addressing the latest advances in the use of light-responsive materials for photobiology have been selected for publication, including phototherapeutic biomaterials proposed for light-induced therapies in cancer, microbial infections, atherosclerosis, aesthetic dentistry treatments (e.g., dental bleaching), and photobiomodulation (PBM) for neurological and neuropsychiatric disorder treatment.

In the design of advanced materials aiming to improve photoactivity efficiency, Lu et al. proposed a dual-activated nanoprodrug for combined chemo-photodynamic therapy of breast cancer based on a glutathione (GSH)-responsive BODIPY photosensitizer and a reactive oxygen species (ROS)-responsive thioketal linker connecting BODIPY and the chemotherapeutic agent camptothecin (CPT). After entering the tumor through passive targeting, the prodrug encapsulated with the amphiphilic polymer DSPE-mPEG2000, is activated by the high tumoral concentration of GSH. Light-triggered ROS from activated BODIPY not only induced apoptosis/necrosis of the tumor cells but also cleaved the thioketal linker to release on-demand CPT, achieving combined photodynamic therapy (PDT) and chemotherapy in mouse mammary carcinoma 4T1, human breast cancer MCF-7 cell lines, and also in 4T1 tumor-bearing mice. The IC50 values were 0.50 μM for the 4T1 cells and 0.63 μM for the MCF-7 cells, attesting for the strong photocytotoxicity exhibited against the tumor cells. Moreover, efficient tumor-targeting and tumor-suppressive effects were observed in 4T1 tumor-bearing mice, without toxicity and side effects, which is of great significance for cancer treatment.

With the aim of promoting breast tumor cell death in situ by photosensitization, Díaz et al. proposed FLTX2, a Tamoxifen derivative endowed with antiestrogenic, fluorescent, and photosensitizer properties, as a selective modulator of estrogen receptors (SERM) for the treatment of estrogen receptor (ER)-positive breast cancer. FLTX2, obtained through the covalent binding of tamoxifen as the ER binding core, 7-nitrobenzofurazan (NBD) as the florescent dye, and Rose Bengal (RB) as a source of ROS, showed a strong absorption in the blue spectral range, associated with the NBD moiety, which efficiently transferred the excitation energy to RB through an intramolecular FRET mechanism, generating superoxide anions that induced concentration- and time-dependent MCF7 apoptotic cell death.

Gangemi et al. proposed the synthesis of a novel bichromophoric system consisting of one subunit of curcumin (donor) and one of BODIPY (acceptor) able to emit in the far-red region, offering a large Stokes shift, capable of limiting light scattering processes. The dyad was encapsulated in small-sized mesoporous silica nanoparticles of MCM-41 (50–80 nm) and tested in human fetal osteoblastic cells (hFOB 1.19) and human bone osteosarcoma epithelial cells (U-2 OS), as models of normal and cancer bone cells, respectively. The bichromophoric system maintained a very efficient photoinduced intercomponent energy transfer even within the hybrid silica system, being located within the cytoplasm without losing brightness, confirming their applicability in bioimaging.

The phototoxicity of psoralen with light of different wavelengths (UVC: λ = 254 nm; UVA: λ = 366 nm) as well as the effect of ionizing radiation (radioisotope Re-188), the emitted beta radiation of which generates Cherenkov light with a yield of 35 photons per decay, was studied by Hübinger et al. Psoralen itself did not show toxic effects on the plasmid DNA or FaDu human cancer cells. After additional treatment with light, a concentration-dependent increase in single strand breaks (SSBs) was visible due to the photochemical activation of psoralen. Conversely, no additional significant Cherenkov-induced phototoxicity was observed when Re-188 was combined with psoralen.

Won Ahn et al. prepared photoactivatable nanocomplexes for treating atherogenic foam cells by encapsulating hydrophobic chlorin e6 (Ce6) within the triple helix structure of β-glucan (Glu) in aqueous solution, with the aim of overcoming the main limitations of the use of Ce6 as a photosensitizer, i.e., its insolubility in water and low selectivity to target cells or tissues. The Glu/Ce6 nanocomplexes were efficiently internalized into foam cells (due to the specific targeting of the dectin-1 receptor) as compared to normal macrophages and they delivered Ce6 into the cytoplasm. The intracellular uptake increased up to 2.6-fold, compared to free Ce6, leading to enhanced PDT effects. In fact, upon NIR laser irradiation, they generated singlet oxygen, inducing significant in vitro photodynamic effects with membrane damage and cell apoptosis.

A comprehensive summary of the contributions of BODIPY to PDT was provided by Malacarne et al. Despite the few molecules approved for PDT in the clinical setting belonging almost exclusively to the porphyrin family [3], the scientific interest towards other photosensitizers, including BODIPY, is exhibiting tremendous growth. This review focused on a series of structural changes made to BODIPY to favor intersystem crossing and further increase ^1^O_2_ production with the final aim of improving cell targeting and/or photoactivity efficiency, pointing out that the absorption and emission features of BODIPY can be modulated by adding suitable substituents to the main chemical structure (e.g., the introduction of heavy atoms, such as bromine and iodine, in the beta positions of the pyrrole ring).

PDT has been shown to be a useful approach not only for cancer treatment but also for treating microbial infection induced by Gram-positive and Gram-negative bacteria, including antibiotic-resistant strains. When the cells being destroyed are microorganisms, this form of therapy is called antimicrobial photodynamic therapy (aPDT) and it shares with “classical” PDT the cooperation of three elements, such as a photosensitizer, light, and oxygen. Interestingly, these components are harmless by themselves but when combined they can lead to the selective destruction of pathogenic cells [4].

The photoinactivation of *Pseudomonas aeruginosa* biofilm by dicationic diaryl-porphyrin was proposed by Orlandi et al. The high binding yield of cationic diaryl-porphyrins (80–100%) could be ascribable to the electrostatic force displayed between negatively charged lipopolysaccharides on the outer layer of the outer membrane and positively charged photosensitizers. Since a mild effect on the formed biofilm was obtained, the results could pave the way for the development of combined strategies to eradicate *P. aeruginosa* biofilms based on dicationic diaryl-porphyrin-mediated aPDT in addition to other antimicrobial approaches.

Higuchi et al. investigated the bactericidal effect of indocyanine green (ICG)-loaded nanospheres coated with chitosan and a diode laser for photodynamic inactivation of a biofilm of *Enterococcus faecalis*, a pathogen of refractory apical periodontitis, an inflammatory lesion causing bone resorption around the apex of teeth. In vitro results showed that antimicrobial photodynamic therapy/photodynamic antimicrobial chemotherapy (aPDT/PACT) can suppress *E. faecalis* in infected root canals with high efficiency (the viable cell counts were reduced by more than 98%) maintaining a temperature rise in the root within a safe range. Morphological observations with SEM confirmed a clear reduction in the biofilm on the dentin block, but the removal was not complete. The authors proposed for future clinical applications, the bactericidal effect should be investigated under changing conditions, such as a smaller interval for the irradiation time and multiple injections of photosensitizer to improve the bactericidal activity.

The influence of incubation time on ortho-toluidine blue (TBO)-mediated aPDT was investigated in vitro against selected *Candida* strains (*C. albicans*, *C. glabrata*, *C. krusei*, *C. parapsilosis*) by Wiench et al. An appropriate incubation time in the aPDT protocol seems to have a great impact on its efficacy, particularly in relation to Candida, due to the size of these cells and the presence of their cell wall. The study pointed out that the most efficient period needed for the uptake of TBO by almost all *Candida* strains was 7–10 min, as confirmed by direct observation by optical microscopy and by evaluation of the efficacy of TBO-mediated aPDT on planktonic cells of these strains.

Amino-functionalized nitrogen-doped graphene quantum dots (amino-N-GQDs) were proposed for aPDT by Kuo et al. as photosensitizers able to generate more ROS than conventional GQDs under 60 s of low-energy (fixed output power: 0.07 W·cm^−2^) excitation exerted by a 670 nm continuous-wave laser. The generated ROS were used to eradicate a multidrug-resistant strain of methicillin-resistant *Staphylococcus aureus* (MRSA), at low energy levels within an extremely short photoexcitation period. Compared with conventional GQDs, the amino-N-GQDs displayed superior optical properties, including stronger absorption and luminescence, a higher quantum yield (0.34), and high stability, contributing to their suitability as contrast probes for biomedical imaging, in addition to their bacteria tracking and localization abilities.

The combination of the use of natural substances with antimicrobial properties and light irradiation at proper light waves, called photodynamic inactivation (PDI), is based on the ability of some natural substances to act as photosensitizers producing bioactive effects under irradiation [5]. Kim et al. demonstrated that fagopyrin F-rich fraction (FFF) separated from *Tartary buckwheat* flower extract when exposed to blue light (BL, 450 nm) produced ROS able to elicit antibacterial photodynamic inactivation (PDI) against *Streptococcus mutans* and its biofilm, which was visually confirmed by confocal laser scanning microscopy (CLSM) and field emission scanning electron microscope (FE-SEM). Interestingly, the PDI effect of FFF against *S. mutans* was similar to curcumin and hypericin and was stronger than riboflavin, although the PDI treatment of FFF was conducted at lower energy fluences of BL and lower concentrations than other photosensitizers.

Some practical applications of PDI are related to food preservation from bacterial contaminants [6]. The development of innovative systems based on natural products and physical methods, such as PDI producing bioactive effects under irradiation, is an emerging and promising research area well reviewed by Munir et al. They explored the antibacterial properties of photoactivated curcumin as a green tool for the preservation of food from bacterial contaminants, since curcumin is a natural antibacterial and effective photosensitizer able to induce photodynamic activation in the visible light range, specifically for blue light.

The great therapeutic potential of photobiomodulation (PBM) therapy in different branches of medicine has been reviewed by Salehpour et al. PBM therapy, namely the application of visible and near-infrared (NIR) light to stimulate cellular processes by changing the biochemical activities of mitochondrial components, is currently applied as a cutting-edge technology in several areas of medicine (i.e., wound healing, dentistry, muscle and tendon repair, dermatology, and neurology). Specifically, this review demonstrated that PBM therapy can serve as a non-invasive neuroprotective strategy for maintaining and optimizing effective brain waste clearance, exerting a neurotherapeutic benefit on glymphatic drainage. The glymphatic drainage system is a waste clearance pathway in the central nervous system devoted to removing toxins and waste metabolites from the brain (i.e., soluble proteins such as amyloid-beta) and its impairment can increase the incidence of neurovascular, neuroinflammatory, and neurodegenerative diseases.

Kim et al. demonstrated that PBM is a promising treatment for neurological and neuropsychiatric disorders, such as autism spectrum disorder (ASD). They used mice exposed to valproic acid (VPA) as a model of ASD to investigate whether PBM treatment during fetal development could attenuate the symptoms of ASD. Their results suggest that the pathological behavioral and histological changes induced by VPA were attenuated by PBM treatment with an 830 nm laser.

One of the molecular mechanisms for the PBM treatment implicates the mitochondrial enzyme, cytochrome C oxidase [7]. With the aim of improving PBM devices and delivery approaches, Amaroli et al. designed a novel handpiece with a flat-top beam profile of irradiation and they compared the beam profile versus a standard handpiece and a fiber probe on mitochondrial activity. They utilized isolated mitochondria and performed treatments at various spots within the beam, namely, the center and left and right edge. The mitochondrial activity was examined by assessing ATP synthesis with the luciferin/luciferase chemiluminescent method as a primary endpoint, while mitochondrial damage was assessed as the secondary endpoint. Their results demonstrated that the novel flat-top beam handpiece enhanced the uniformity of the PBM treatments and can improve the rigor and reproducibility of PBM clinical outcomes.

Since several experimental parameters and factors influence the lifetime of specific fluorophores, different values of fluorescent lifetimes are sometimes found in the literature for the same fluorophore depending on which detection and excitation scheme is used. To clarify this controversy, Kellerer et al. reported a comprehensive and rigorous investigation of parameters influencing fluorescence lifetime imaging microscopy in the frequency domain (FD) and time domain (TD), illustrated by phasor plot analysis. These two most common techniques were implemented in one single microscopy setup and applied to a variety of fluorophores under different conditions of pH, temperature, concentration, and solvent polarity. All the studied parameters fall within two categories (setup-dependent and sample-dependent) and both FD and TD techniques produce reliable and consistent data revealing which of the tested parameters has the strongest influence on the fluorescence lifetime. In addition, their results suggested which technique is most suitable for which research task and how to perform the experiment properly to obtain consistent fluorescence lifetimes.

An in vitro 3D tumor model composed of human melanoma cells and the microalgae *Chlamydomonas reinhardtii*, both seeded into a collagen scaffold, was presented by Holmes et al. as a representative and reproducible model for studying photosynthetic tumor oxygenation. Their results, surprisingly, demonstrated that, although the investigated conditions significantly differ from the optimal microalgae culture settings in key aspects such as temperature, medium composition, and the presence of tumor cells, these conditions do not seem to inhibit the intrinsic ability of the microalgae to provide significant amounts of oxygen in the presence of light. This reproducible and easy-to-use model can be used as a platform for studying the role of other key cell types in the tumor microenvironment and also for studying the antitumoral effects of ROS-dependent photodynamic, chemo-, immuno-, and radiotherapy.

De Plano et al. evaluated the role of capsid rearrangement in engineered phages of M13 in protecting viral DNA and peptide bonds from damage induced by UV-C radiation. The study was performed on two M13 engineered phage clones (P9b and 12III1 phages expressing 9 or 12 additional amino acids in N-terminal end of pVIII capsid protein) and their resistance to environmental stresses, such as UV-C radiation and hydrogen peroxide, was compared to M13 wild-type vector (pC89). Only P9b displayed an increase in resistance against H_2_O_2_, whereas both clones acquired UV-C resistance due to a reorganization of interactions in the capsid for an increase in H-bond and steric interactions, which in turn depended on the presence of the foreign peptide sequence. These results could help us to understand the molecular mechanisms involved in the stability of new virus variants, useful for developing effective protocols for virus inactivation.

Thacker et al. evaluated the efficacy and cytotoxicity of a dental bleaching gel composed of calcium peroxide (CaO_2_) as an active ingredient, visible-light-activating nitrogen-doped titanium dioxide as a photocatalyst, and methylcellulose as the thickener. The study was performed in vitro on bovine teeth stained with coffee and black tea stain solution that were subjected to one minute of visible light irradiation during each bleaching time. The gel demonstrated at neutral pH an efficient bleaching effect with a gradual increase in brightness (ΔL) and color difference (ΔE), without cytotoxicity upon exposure to 3T3 cells. Moreover, the proposed gel allowed for the avoidance of potential side effects usually caused by a highly concentrated hydrogen peroxide-based dental bleaching procedure.

The nineteen articles published in our Special Issue demonstrate the growing interest in the use of light-responsive materials for applications in the field of photobiology and in related biomedical areas in recent years. Materials for photobiology not only were realized by the utilization of novel molecular components for light-responsive processes in living organisms, but also comprised living matter itself in which light-induced processes occur. We hope to supply our readers with some representative and useful snapshots of actual research, perhaps providing the inspiration to push developments in this field a step further. We acknowledge all the contributors of the Special Issue and the Editorial Board of *Int. J. Mol. Sci.* for their support.
